# Research efficacy of gaseous ozone therapy as an adjuvant to periodontal treatment on oxidative stress mediators in patients with type 2 diabetes: a randomized clinical trial

**DOI:** 10.1186/s12903-023-02985-1

**Published:** 2023-05-11

**Authors:** Biagio Rapone, Elisabetta Ferrara, Erda Qorri, Francesco Inchingolo, Gaetano Isola, Paola Dongiovanni, Gianluca Martino Tartaglia, Antonio Scarano

**Affiliations:** 1 Interdisciplinary Department of Medicine, “Aldo Moro” University of Bari, Bari, 70121 Italy; 2 Department of Medical, Oral and Biotechnological Sciences, University G. d’Annunzio, Chieti, 66100 Italy; 3 Dean Faculty of Medical Sciences, Albanian University, Bulevardi Zogu I, Tirana, 1000 Albania; 4 Department of General Surgery and Medical Surgery Specialties, School of Dentistry, University of Catania, 95123 Catania, Italy; 5 General Medicine and Metabolic Diseases, Fondazione IRCCS Ca’ Granda Ospedale Maggiore Policlinico, Pad. Granelli, via F Sforza 35, Milan, 20122 Italy; 6 UOC Maxillo-Facial Surgery and Dentistry, Ospedale Maggiore Policlinico, Fondazione IRCCS Ca Granda, Milan, 20122 Italy; 7 Department of Biomedical, Surgical and Dental Sciences, University of Milan, Milan, 20122 Italy; 8 Department of Innovative Technologies in Medicine and Dentistry, University of Chieti-Pescara, Chieti, 66100 Italy

**Keywords:** Periodontitis, Periodontal inflammation, Type 2 diabetes, Oxidative stress, Antioxidant status, pro-oxidant status

## Abstract

**Background:**

Chronic inflammation and cumulative oxidative stress have been theorized as two common pathways of the interconnection between periodontitis and diabetes. Improvement in oxidizing status has been demonstrated in periodontal patients with diabetes treated with proper non-surgical periodontal treatment. In addition to periodontal treatment, Gaseous ozone therapy has been reported to possess anti-inflammatory properties and the ability to stimulate the endogenous antioxidant defence mechanism. To date, the antioxidant effect of gaseous ozone, in addition with periodontal treatment in diabetic patients, has been examined in only one study. The aim of this study was to determine the efficacy of gaseous ozone therapy as an alternative approach to supporting non-surgical periodontal therapy (NSPT), aimed at improving antioxidant machinery and interfering with ROS production on plasma levels in diabetic individuals diagnosed with moderate or severe periodontitis.

**Methods:**

One hundred and eighty patients with periodontitis and type 2 diabetes mellitus were randomly assigned to receive non-surgical periodontal treatment (NSPT) plus gaseous ozone therapy (A) NSPT alone (B). Clinical and periodontal parameters -Bleeding on probing (BOP), Periodontal pocket depth (PPD), and Clinical attachment Level (CAL)- and plasma levels of oxidant-antioxidant (TOS- TAOS) levels, glutathione (GSH), and malondialdehyde (MDA) were recorded at baseline and at 3- (T1) and at 6-months (T2) after treatment.

**Results:**

Both treatments were efficacious in reducing clinical parameters. However, there were no significant differences regarding oxidative stress parameters in group A compared to group B.

**Conclusions:**

In the present study, gaseous ozone therapy did not enhance the effect of periodontal treatment in reducing oxidative stress in plasma levels of periodontitis patients with type II diabetes.

**Trial registration:**

The study was registered with ISRCTN1728169 (23/07/2022).

**Supplementary Information:**

The online version contains supplementary material available at 10.1186/s12903-023-02985-1.

## Background

Over the past decade, the interactions between periodontal inflammation and systemic reactions have become better understood, and this has motivated the recognition that periodontitis has pathogenic effects on diabetes, depending on biological circumstances and a highly orchestrated immune response [1–3]. Periodontitis is one of the most common infectious-inflammatory diseases involving the periodontium, distinguished by the gradual and progressive deterioration of periodontal tissues with concomitant structural and functional changes [4]. The paroxysmal host response to the etiologic agents and its products is the primary determinant of the periodontal reaction, mediated through inflammatory pathways that are regulated with support of environmental and epigenetic mechanisms [5, 6]. Periodontitis is significantly related to a reduction in total antioxidant status (TAS TAOS) and an increase in reactive oxygen metabolites [7–9]. The immune response and oxidative stress progressively catalyse degenerative changes that ultimately affect periodontal architecture. Under physiological conditions, equilibrium is maintained by the balance between ROS production and ROS scavenging [10–12]. Conversely, oxidative stress occurs when the antioxidant protective system becomes pathologically unbalanced [13]. ROS are the most abundant intracellular oxidizing substances and act as essential mediators and signalling molecules in many biological processes [14, 15]. The biological task of ROS is dichotomous, contributing to physiologic and pathological conditions. In the physiological state, the proteolytic-anti proteolytic equilibrium exists, and ROS can act as a second messenger in innate and adaptive immune cells that regulate the activities of proteins specific to the maintenance of biological functions [16–18]. In the pathological condition, the enhanced generation of free radicals is observed, responsible for the modification of proteolytic -antiproteolytic balance [19]. The rise in ROS levels is crucial to orchestrate the peripheral response to chronic periodontal infection by promoting hyperactivation of the inflammatory cascade [20–22]. Periodontitis occurs three times more often in diabetic patients than in healthy individuals, although there is considerable heterogeneity in patients in terms of phenotypic expression [23–25]. Common factors clarify the increased risk of increased inflammation. Clinical studies examining the association between periodontitis and diabetes have indirectly demonstrated the role of periodontal inflammation, by IL-6 -, TNF-α-, IL-1β-, and C-reactive protein-induced pro-inflammatory process [26]. Oxidative stress and chronic inflammation in humans promote the dysregulation of adipokines (e.g., leptin, TNF-α, IL-6, adiponectin, etc.) synthesis and secretion which can lead to secondary metabolic disorders including insulin resistance [27]. The phenomenon of low-grade inflammation related to periodontitis results in a chain of events leading to an increase levels in oxidative stress and the release of free radicals in diabetes. Oxidative stress plays a pivotal role in the development of diabetes complications by causing excess mitochondrial hydrogen peroxide emission in the skeletal muscle, altering redox status and promoting insulin resistance [23–25, 28]. Disruption of appropriate physiological function and debilitation of the antioxidant defence system can advance the evolution of diabetes by increasing the frequency of oxidative damage to erythrocytes [29–31]. The supraphysiological production of reactive oxygen and nitrogen species (ROS/NOS) and ineffective antioxidant defence modify the structural and mechanical properties of erythrocytes, which result in changes in mechanical properties, elevate susceptibility to the mechanic and osmotic shock, and damaging of N-terminal of band-3 proteins (B3p) by modifying the anion exchange process, and glycophorin, resulting in decline in the cell’s surface area [32]. Erythrocyte imbalance can make them dysfunctional and impede efficient tissue oxygenation.

Proper periodontal treatment has beneficial effects on the reduction of oxidative stress [33–37]. Because of its strong antimicrobial and anti-inflammatory properties, ozone is frequently employed in dentistry [38–40]. Thus, it was tempting to speculate that gaseous ozone therapy add-on periodontal treatment might be effective in reducing oxidative stress outcomes in diabetic patients. We speculated that non-surgical periodontal therapy (NPTS) in combination with ozone therapy would counteract the oxidative stress of patients with diabetes with periodontitis.

## Methods

### Study setting and Ethics

**Fig. 1:**
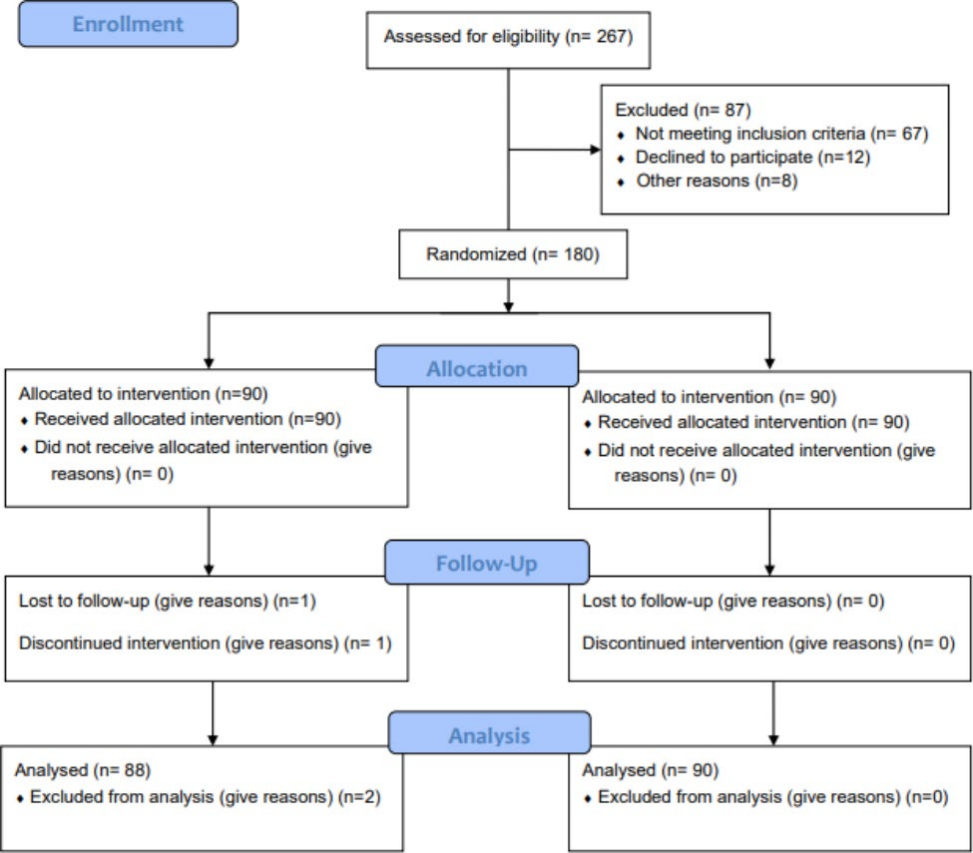
Participant flow diagram

Between June 2018 and September 2019, this prospective, controlled, one-blind, randomized clinical trial enrolled 180 diabetic subjects who were outpatients at the Clinic of Periodontology of the Faculty of Dental Medicine of Albanian University. All participants provided informed written consent prior to patient enrolment and randomization. The study protocol was approved by the Institutional Review Board at Albanian University (reference number 2018/224) and was performed in compliance with good clinical practice and followed the ethical standards established in the Declaration of Helsinki. The study was registered with ISRCTN17281691. Patients consenting to participate in the study were randomly assigned to one of two treatment groups. The study design followed the CONSORT guidelines [41]. Figure 1 shows the flow diagram of the study’s progress through all phases.

### Study population

Eligibility criteria for the study were assessed at in-person screening visits involving laboratory blood assessment, comprehensive clinical periodontal examination, and detailed medical history. To be eligible to participate in this study, patients had to meet the T2DM diagnostic criteria recommended by the American Diabetes Association (ADA) in 2020: FPG ≥ 126 mg/dL (7.0 mmol/L), or 2-h PG ≥ 200 mg/dL (11.1 mmol/L) during OGTT, or HbA1C ≥ 6.5% (48 mmol/mol), or a random plasma glucose ≥ 200 mg/dL (11.1 mmol/L).

Inclusion criteria included poorly controlled (HbA1c > 7%) T2DM, adult patients (age ≥ 18 years); having no significant comorbidity, being not involved in any trial/study related to diabetes and periodontitis during last 12 months and able to attend regular visits, the presence of at least 16 natural teeth that were distributed in all four quadrants. Among the teeth, at least six had to exhibit one site with a pocket depth (PD) ≥ 5 mm at baseline. Periodontitis was graded according to the diagnostic criteria co-presented the American Academy of Periodontology (AAP) and the European Federation of Periodontology (EFP) in the World Workshop on the Classification of Periodontal and Peri-Implant Disease and Conditions [42]. Key exclusion criteria included gestational and type 1 diabetes mellitus and cognitive impairments, pregnancy and lactation, subjects who had received periodontal therapy or taken an antibiotic during the previous six months.

### Study enrolment and randomization

Randomizing software was used to randomly allocate patients on a 1:1 ratio to non-surgical periodontal therapy (NSPT) + Ozone group, and NSPT. The patients in group A (n = 90) received non-surgical periodontal treatment plus gaseous ozone therapy. Patients in Group B (n = 90) received non-surgical periodontal treatment. All follow-up periodontal evaluations were performed by the same two calibrated operators. The unblinded clinician determined the treatment for the subjects. Participants were not blinded because the motivation session was carried out according to the allocation. Prior to a consultant appointment, patients visit the department of Dentistry for periodontal clinical parameters assessments. During this phase, patients were provided with a general information sheet. Patients who met study eligibility criteria signed the informed consent, after which they were enrolled into the research study and, randomized to either NSPT + Ozone treatment (Test Group, A) or the control treatment (Control Group, B) according to the randomization sequence. Secondly, eligible patients were actively involved in the research study by screening phase. Subjects provided informed consent prior to initiation of any study procedures. Individuals were excluded if they presented declined cognitive function and unable to make informed consent, underwent periodontal treatment within least 12 months, and systemic antibiotic therapy within the last 6 months, pregnancy, or breast feeding, having uncontrolled hypertension, history of heart disease or stroke which might interfere with systemic antioxidative status.

### Periodontal parameters

A comprehensive periodontal charting and intraoral examination were performed for all participants, and the periodontal status was examined by measurements of prior to and after the treatment in both groups and encompassed measurements of the following clinical periodontal parameters: clinical attachment loss (CAL), probing pocket depth (PPD), and bleeding on probing (BOP). Bleeding was determined using a periodontal probe (PCP UNC-15, Hu-Friedy, Chicago, IL) that flowed along the soft tissue wall of the entrance of the gum fissure and parallel to the long axis of the tooth in the facial/ lingual sites and directed towards the column in the interproximal sites; CAL was measured as the distance from a fixed reference point (cementoenamel junction, CEJ) to base of pocket, whereas PPD was measured as the distance between the free gingival margin and the clinically base of each periodontal pocket. Full mouth series (FMS) radiographs were obtained prior all treatments for all subjects to confirm alveolar bone level. Clinical parameters assessment was performed by measuring on six sites of teeth (mesial, median, and distal points at buccal and palatal aspects) [43]. Additionally, the periodontal inflamed surface area (PISA) was assessed to quantify periodontitis severity. Clinical measurements were performed by the same blind and calibrated investigators using the same type of periodontal probe (PCP UNC-15, Hu-Friedy, Chicago, IL) with a mild probing force corresponding to approximately 25 N. About intra-examiner reproducibility, the intraclass correlation coefficient (ICC) was used to assess the agreement of findings, based on the PD measurements. The ICC was calculated using the R package irr. An intra-examiner agreement of 0.989 (95% CI, 0.916–0.999) was found.

### Monitoring procedures-data collection

Medical history, and current medication were collected, and a comprehensive periodontal examination at study enrolment was performed. According to the local laboratory’s standard procedure, routine laboratory parameters were analyzed from venous blood samples. All study visits occurred in the Outpatient Laboratory of the Center for diabetes control. Each participant completed three inpatient study visits for periodontal and oxidative status assessment: a baseline visit, 3-month visit, and 6-month visit. During the initial evaluation, oxidative status assessment was carried out prior to treatment. This protocol was repeated at the three- and six-month re-evaluation to assess post-treatment oxidant and antioxidant parameters.

### Interventions

The interventions in groups A and B comprised full-mouth scaling and root surface debridement completed within 6 weeks, operating one scaling session of 45 min, 4 sessions of root planning. The scaling session consisted of ultrasonic supra- and subgingival debridement of soft and hard deposits of bacterial plaque and calculus. After completing the measurements, all pockets with PPD ≥ 4 mm were scaled, and root planed under local anesthesia with Gracey curettes (Hu-Friedy®) and ultrasonic instruments (Piezon® 250; Electro Medical Systems SA) by the same clinicians. After all mechanical treatment sessions, in group A, the patients rinsed for 1 min with 15 mL of ozonized oil mouthwash (Ialozon Blu; Gemavip, Cagliari, Italy), and after mechanical instrumentation. Patients in group B did not received mouthwash. Oral hygiene instructions were given and scaling and root surface debridement was performed with piezoelectric and hand instruments. Upon leaving each session, participants were subjected to oral hygiene instruction by emphasizing the use of manual or electric toothbrushes twice daily, tongue cleaning via brushing or scraping to reduce potential pathogenic organisms residing on the dorsum of the tongue, interproximal cleaning to disrupt plaque formation. We established sequential treatment plan with three steps of gaseous ozone therapy after the instrumentation by ultrasonic devices. As our previous research [44], an ozone generator (Ozone DTA, Sweden & Martina Company; Carrara San Giorgio, Veneto, Italy), was employed, according to manufacturer’s instructions, as follows: Step (1) 2-min rinse with ozonated water at a ratio of 1:3; full-mouth decontamination; topical irrigation with ozonated water; 1–2 cycles of ozone gas at 8–10 power in correspondence of pathological pockets, under local anesthesia. Step (2) Quadrant root planning; 2-min rinse with ozonated water at a ratio of 1:3; Deplaquing; 1–2 cycles of ozone gas at 8–10 power in correspondence of pathological pockets for each quadrant, under local anesthesia. Step (3) Maintenance: 2-min rinse with ozonated water at a ratio of 1:3; Deplaquing; 1–2 cycles of ozone gas at 4–5 power in correspondence of pathological pockets for all quadrants, two weeks after completion of treatment. Patients of Group A were instructed to rinse with commercial mouthwash (Ialozon Blu; Gemavip, Cagliari, Italy) twice a day after brushing. All patients were instructed to abstain from use of all types of commercial mouthrinses.

### Supportive periodontal treatment

Supportive periodontal therapy (SPT) was performed at 3 and 6 months and consisted of reinforcement of patient-performed oral hygiene and detailed monitoring of the periodontal tissues (examination of extraoral and intraoral soft tissues; dental examination and radiographic review; evaluation of the patient’s oral hygiene performance; periodontal evaluation and risk assessment). In addition, supragingival and subgingival removal of bacterial plaque and calculus was performed, and gaseous ozone therapy was applied as a maintenance treatment to each session for patients of group A. [43].

### Plasma levels of oxidative status assessment

Oxidative stress assessment of participants was performed at baseline, 3 and months follow-up. Serum samples were obtained by centrifugation (1500 × g, 10 min) of the collected blood samples and then kept at -80 °C until the GSH, MDA, TAOS, and TOS parameters were analyzed. Then, they were transferred to the University of Albanian for analyses. Serum concentrations of glutathione (GSH), and malondialdehyde (MDA) were measured by employing a Sciex API 3000 triple quadrupole mass spectrometer as described previously [31]. The concentration of MDA was calculated using an extinction coefficient of MDA–TBA complex, which is 1.56 × 105 M − 1 cm − 1 and the results were expressed as nmol MDA/mg protein. Total glutathione content was measured according to the method described by Beutler et al. [40] using glutathione reduced colorimetric method [42]. Results were expressed as mg/dl. The results were shown as nmol 2HO Eq/mg for TOS, and µmol Trolox Eq/mg for TAOS. The reference range for TOS and TAS were 5.54– 21.38 µmol 2HO Equiv./L, and 1.20– 1.81 mmol Trolox equiv./l, respectively.

### Statistical analysis

All the analyses were performed with SPSS for Windows (Version 13.0, SPSS Inc., Chicago, IL, USA). A multivariate analysis of covariance (MANCOVA) was conducted to assess if there were significant differences in the linear combination of TOS, TAOS, GSH, MDA, PPD, CAL, and BOP at baseline and during follow-up between the levels of severity of periodontitis and treatment after controlling for age, smoking status, and sex. Before conducting MANCOVA, Pearson’s correlation was performed between all the dependent variables to test the MANCOVA assumption that the dependent variables would be correlated in the moderate range [33]. A significant pattern of correlation was observed amongst the following dependent variables, suggesting the appropriateness of MANCOVA. Additionally, the Box’s M value of 138.53 was associated with a *p-*value of 0.008, indicating that the covariance matrices for each group were not statistically significantly different from one another and that the assumption was met based on Huberty and Petoskey’s guidelines [36]. When an effect was significant in MANCOVA, one-way analysis was used to discover which dependent variables had been affected. Statistical testing of the model was performed by the Fisher’s statistical test for analysis of covariance (ANCOVA). This approach evaluated an associated probability (p-value) for each term of the regression model. For a given confidence level (95% in our case), an effect with p-value higher than 0.05 was not considered statistically significant on the output variable [44]. Fixed variables were treatment groups (test, A, or control, B), and degree of severity of periodontitis (moderate or severe). Each participant was included as a random effect. To test for confounders, an a priori set of variables were considered as covariates: age, sex, and smoking status (yes/no) at baseline. Any covariate exerting a change in effect estimate of at least 10% was included in the analysis as a confounder. The only confounder noted was smoking for analysis of oxidant status and was therefore integrated as a confounder in all analyses [45]. Residuals from the outcome variables were reviewed about model assumptions. The assumption of normality was assessed by plotting the quantiles of the model residuals against the quantiles of a Chi-square distribution, and all were normally distributed. Homoscedasticity was evaluated by plotting the residuals against the predicted values. Mauchly’s test was used to assess the assumption of sphericity. Mahalanobis distances were calculated and compared to a χ2 distribution to identify influential points in the residuals. An outlier was defined as any Mahalanobis distance that exceeds 22.46, the 0.999 quantile of a χ^2^ distribution with 6 degrees of freedom. There were no outliers detected in the model. A correlation matrix was calculated to examine multicollinearity between the dependent variables. The correlation matrix is presented in Appendix 1.

*Homogeneity of regression slopes.* As shown in Appendix 2, the assumption for homogeneity of regression slopes was assessed by rerunning the MANCOVA, but this time including interaction terms between each independent variable and covariate. If the model with the covariate interaction terms explains significantly more variance than the original MANCOVA model, then there were significant interactions between the covariates and independent variables. The model with covariate-independent variable interactions did not explain significantly more variance for all dependent variables than the original model, F(72, 120) = 0.74, p = .914. This implies that none of the covariates interacted with the independent variables and the assumption of homogeneity of regression slopes was met [46].

*Post-hoc.* To further examine the effects of the severity of periodontitis and treatments on all dependent variables at baseline and during follow up controlling for age, smoking status, and sex, an analysis of covariance (ANCOVA) was conducted for each dependent variable [47].

A multinomial regression model was conducted and compared to the null model for each pair of categorical covariates and independent variables to assess independence (Appendix 2). There were no significant models for any combination of covariates and independent variables based on an alpha of 0.05, indicating the assumption of independence between covariates and independent variables was met [48]. Categorical variables are summarized using frequencies and percentages. Continuous variables with normal distributions are presented as mean (± standard deviation); continuous variables with non-normal distributions are presented as medians and interquartile ranges. Differences in baseline characteristics among the groups were examined using the Pearson’s chi-square test or Fisher’s exact test for categorical variables and analysis of variance or Kruskal-Wallis test for continuous variables. The primary endpoints were the mean TOS, TAOS, GSH, and MDA at 3, and 6 months, but PPD, CAL, and BOP were also subject to secondary analyses. Likewise, dichotomous outcomes were tested using logistic regression analysis of the effect of the two interventions and their interaction term on the outcome. The significance level was set at P < .05.

### Sample size calculation

The power analysis of the study was performed on the primary outcomes TOS and TAOS. To detect a change of 20% between groups with 90% power and α = 0.05, a sample size of 50 patients per group was needed and to account for dropouts 180 patients were recruited. Primary outcomes in this report were TOS and TAOS plasma concentration. Secondary outcomes were periodontal parameters and HbA1c levels.

## Results

Out of the 235 patients screened, 180 were enrolled. Two patients were withdrawn from treatment because of cardiomyopathy (1) and pregnancy (1) during the follow-up. Overall, one-hundred-two participants were female (58.8 ± 10.8 years), and 78 (56.9%) were men (54.9 ± 16.5 years). The intervention period lasted for 6 months (8.12 median, ± 13.7 weeks). Baseline characteristics of both trial cohorts were similar. The clinical and biochemical characteristics between groups at baseline are summarized in Table 1.

**Table 1:** Comparisons of the baseline characteristics between participants assigned to groups

	Group Test (A)	Group Control (B)	*p*
***n***	90	90	
	M (SD)	M (SD)	
**Age (years)**	53.8 ± 10.8	61.9 ± 16.5	0.421
**Duration of Diabetes (years)**	7.3 ± 4.8	8.1 ± 5.9	0.467
**BMI (kg/m** ^**2**^ **)**	24.3 ± 3.8	25.5 ± 4.7	0.037*
**Systolic BP (mmHg)**	139 ± 32	143 ± 21	0.076
**Diastolic BP (mmHg)**	94 ± 10	94 ± 12	0.856
**FPG (mmol/l)**	10.7 ± 4.8	9.3 ± 5.2	0.458
**HbA**_**1c**_ **(%)**	8.7 ± 1.6	7.86 ± 1.3	0.085
Fasting insulin (µU/ml)*	11.25×/÷2.27	11.70×/÷2.34	0.566
**TC (mmol/l)**	5.6 ± 1.1	5.8 ± 1.4	0.159
**TG (mmol/l)**	1.49×/÷1.95	1.52×/÷1.88	0.957
**LDL-C (mmol/l)**	3.6 ± 1.0	3.6 ± 1.1	0.456
**HDL-C (mmol/l)**	1.19 ± 0.31	1.34 ± 0.34	< 0.001*
**BOP (%)**	59.61 ± 0.75	62.76 ± 0.16	< 0.001*
**PPD (mm)**	5.8 ± 0.56	5.98 ± 0.56	< 0.001*
**CAL (mm)**	5.49 ± 0.89	6.32 ± 0.21	< 0.001*
**Smoker (yes/no)**	60.5%	47.5%	< 0.001*

The main effect for degree of periodontitis’ severity was significant, F(15, 19) = 2.32, p = .043, η2p = 0.65, suggesting the linear combination of each dependent variable was significantly different between the levels of severity of disease after controlling for age, smoking status, and sex. The main effect for treatment was significant, F(15, 19) = 4.73, p < .001, η2p = 0.79, suggesting the linear combination of all dependent variables was significantly different between the levels of treatment after controlling for covariates. The covariates, age and smoking status, were not significantly related to dependent variables at each time point, F(15, 19) = 1.61, p = .163, η2p = 0.56, and F(15, 19) = 0.77, p = .695, η2p = 0.38 respectively. The covariate, sex, was not significantly related to each dependent variable, F(15, 19) = 0.93, p = .551, η2p = 0.42. Table 2 shows the results of the MANCOVA.

**Table 2:** MANCOVA Results for each dependent variable by severity of periodontitis and treatment while controlling for the covariates age, smoking status, and sex

Variable	Pillai	F	df	Residual df	p	η_p_2
**severity_of_periodontitis**	0.647	2.320	15	19	0.0427	0.647
**treatment**	0.789	4.726	15	19	9.710 × 10^− 04^	0.789
**age**	0.559	1.608	15	19	0.163	0.559
**smoking_status**	0.377	0.767	15	19	0.695	0.377
**sex**	0.423	0.929	15	19	0.551	0.423

The ANCOVA results were insignificant, F(5, 34) = 0.77, p = .575, indicating the differences among the degree of degree of periodontitis’ severity and treatment were insignificant (Table 3). The main effect for degree of periodontitis’ severity was not significant, *F*(1, 34) = 0.63, *p* = .435 for TOS at T0, and the main effect for treatment was not significant, respectively *F*(1, 34) = 0.87, *p* = .357.

**Table 3:** Analysis of Variance Table for each dependent variable at T0, T1, and T2

TOS T0	*SS*	*df*	*F*	*p*	η_p_2	
**severity_of_periodontitis**	35.92	1	0.63	0.435	0.02	
**treatment**	50.06	1	0.87	0.357	0.02	
**age**	63.96	1	1.11	0.299	0.03	
**smoking_status**	86.56	1	1.51	0.228	0.04	
**sex**	21.40	1	0.37	0.546	0.01	
**Residuals**	1,952.85	34				
**TOS T1**						
**severity_of_periodontitis**	4.29	1	0.10	0.752	0.00	
**treatment**	182.88	1	4.31	0.045	0.11	
**age**	51.72	1	1.22	0.277	0.03	
**smoking_status**	77.82	1	1.84	0.184	0.05	
**sex**	13.52	1	0.32	0.576	0.01	
**Residuals**	1,441.81	34				
**TOS T2**						
**severity_of_periodontitis**	0.01	1	0.00	0.985	0.00	
**treatment**	262.88	1	8.37	0.007	0.20	
**age**	59.31	1	1.89	0.178	0.05	
**smoking_status**	51.58	1	1.64	0.209	0.05	
**sex**	10.84	1	0.35	0.561	0.01	
**Residuals**	1,067.86	34				
**TAOS T0**						
**severity_of_periodontitis**	0.04	1	0.60	0.445	0.02	
**treatment**	0.05	1	0.83	0.369	0.02	
**age**	0.0002	1	0.00	0.959	0.00	
**smoking_status**	0.04	1	0.58	0.453	0.02	
**sex**	0.005	1	0.07	0.789	0.00	
**Residuals**	2.23	34				
**TAOS T1**						
**severity_of_periodontitis**	0.04	1	0.60	0.445	0.02	
**treatment**	0.05	1	0.78	0.369	0.02	
**age**	0.0001	1	0.00	0.959	0.00	
**smoking_status**	0.03	1	0.58	0.453	0.02	
**sex**	0.005	1	0.07	0.789	0.00	
**Residuals**	2.17	34				
**TAOS T2**						
**severity_of_periodontitis**	0.02	1	0.41	0.569	0.01	
**treatment**	0.21	1	3.39	0.068	0.01	
**age**	0.008	1	0.02	0.912	0.00	
**smoking_status**	0.02	1	0.29	0.587	0.01	
**sex**	0.02	1	0.35	0.565	0.01	
**Residuals**	1.79	31				
**GSH T0**						
**severity_of_periodontitis**	0.013	1	0.41	0.611	0.01	
**treatment**	0.18	1	3.78	0.092	0.01	
**age**	0.0019	1	0.02	0.780	0.08	
**smoking_status**	0.01	1	0.31	0.591	0.00	
**sex**	0.01	1	0.28	0.545	0.01	
**Residuals**	0.89	37				
**GSH T1**						
**severity_of_periodontitis**	0.02	1	0.35	0.559	0.01	
**treatment**	0.17	1	3.45	0.072	0.09	
**age**	0.0010	1	0.02	0.890	0.00	
**smoking_status**	0.02	1	0.33	0.571	0.01	
**sex**	0.02	1	0.40	0.534	0.01	
**Residuals**	1.69	34				
**GSH T2**						
**severity_of_periodontitis**	0.01	1	0.35	0.559	0.01	
**treatment**	0.15	1	2.89	0.067	0.08	
**age**	0.0011	1	0.03	0.875	0.01	
**smoking_status**	0.02	1	0.33	0.571	0.00	
**sex**	0.01	1	0.40	0.534	0.00	
**Residuals**	1.76	32				
**MDA T0**						
**severity_of_periodontitis**	0.02	1	0.35	0.559	0.01	
**treatment**	0.19	1	3.69	0.081	0.087	
**age**	0.0012	1	0.019	0.890	0.01	
**smoking_status**	0.018	1	0.31	0.571	0.01	
**sex**	0.01	1	0.38	0.534	0.01	
**Residuals**	1.54	34				
**MDA T1**						
**severity_of_periodontitis**	0.02	1	0.35	0.559	0.01	
**treatment**	0.17	1	3.45	0.072	0.09	
**age**	0.0010	1	0.02	0.890	0.00	
**smoking_status**	0.02	1	0.33	0.571	0.01	
**sex**	0.02	1	0.40	0.534	0.01	
**Residuals**	1.69	34				
**MDA T2**						
**severity_of_periodontitis**	0.02	1	0.35	0.559	0.01	
**treatment**	0.17	1	3.45	0.072	0.09	
**age**	0.0010	1	0.02	0.890	0.00	
**smoking_status**	0.02	1	0.33	0.571	0.01	
**sex**	0.02	1	0.40	0.534	0.01	
**Residuals**	1.69	34				

Table 3 shows that there were not statistically significant differences between the values of TOS at baseline among the values of degree of periodontitis’ severity and treatment. The main effect for the degree of periodontitis’ severity was not significant, *F*(1, 34) = 0.10, *p* = .752, indicating there were no significant differences of TOS at T1 by severity of periodontitis levels. However, there were statistically significant differences for treatment, F(1, 34) = 4.31, p = .045, ηp2 = 0.11, indicating significant differences in TOS at 3 months (T1) by treatment levels. Regarding the TOS levels at 6 months (T2) after treatment, the results of the ANCOVA were not significant, F(5, 34) = 2.31, p = .065, indicating the differences among the degree values of periodontitis’ severity and treatment were not relevant. No statistically significant differences among the degree of periodontitis’ severity and treatment were found for TAOS at T1, with *F*(1, 34) = 0.60, *p* = .445, and *F*(1, 34) = 0.83, *p* = .369 respectively. Regarding TAOS at 6 months (T2), both groups experienced similar values. Severity of periodontitis did not affect results, *F*(1, 34) = 0.35, *p* = .559 by degree of periodontitis’ severity levels. No statistically significant changes were observed in the TOS and TAOS oxidative stress markers in either group (*F*(1, 34) = 3.45, *p* = .072). This is consistent with the results regarding MDA and glutathione levels which states that there are not statistically significant differences among groups (*p* = .086). The results of BOP at T2 were significant, *F*(5, 33) = 2.80, *p* = .032 among the values of severity of periodontitis and treatment. The main effect for severity of periodontitis was *F*(1, 33) = 11.11, *p* = .002, η_p_2 = 0.25, indicating there were significant differences in BOP at T2 by severity of periodontitis levels *F*(1, 33) = 3.69, *p* = .063. No significant differences of BOP at T2 by treatment levels were revealed. There were significant differences in CAL at T2 by severity of periodontitis and treatment while controlling for age, smoking status, and sex. The detailed ANCOVA results are reported in Appendix 3.

## Discussion

[4, 8, 11, 37] The number of experimental studies on the molecular and physiological basis of the interaction between periodontitis and diabetes has grown considerably in recent years [31–33]. It has long been understood that individuals with diabetes are significantly more prone to develop periodontitis than healthy subjects, and vice versa. However, inflammation alone is not enough to stratify the risk for both diseases. Recently, it has been reported that in diabetic patients with periodontitis, oxidative damage could also be dependent on periodontal condition [4, 8, 11]. Periodontitis reflects unsuccessful redox homeostasis through indirect mechanisms which impact the intrinsic antioxidant defence system capacity. The metabolic flux regulation of ROS caused by chronic periodontal inflammation leads to a state of moderately increased levels of intracellular ROS [21, 24]. In line with the theory of the bi-directional relationship between periodontitis and diabetes, many researchers have identified oxidative stress as a key factor contributing to this correlation. A dominant view is that an imbalance between reactive oxygen species (ROS) production and elimination by antioxidant defense mechanisms exerts significant negative pressure. Moreover, chronic periodontal inflammation induces the oxidative burst and is significantly associated with upregulated expression of GSH and MDA levels, markers of oxidative stress [37]. Therefore, it has been alleged that periodontal disease probably contributes to the oxidative stress associated with diabetes and chronic inflammation. Mounting evidence has signaled oxidative stress as a core pathophysiology in the development of complications of diabetes [31]. Indeed, oxidative stress plays a critical role in diabetes, and hyperglycemia decreases enzyme activities of superoxide dismutase (SOD) and glutathione (GSH) synthesis, likely via glycation [17]. The effects of long-term exposure to increased levels of pro-oxidant factors exacerbate the outcomes, by triggering a negative chain reaction which affects processes involving the maintenance of homeostasis, as well as a wide variety of cellular functions (mitochondrial dynamics and function, structural modification of cellular proteins and the alteration of their functions, nuclear and mitochondrial DNA dysfunction, lipids, and proteins damage) [40]. No direct causal link has been established between oxidative stress periodontitis-induced and diabetes, and the exact mechanism remains elusive and is not yet entirely clear [31]. Ozone therapy in diabetes has been widely studied because of the link between diabetic nephropathy and oxidative stress, and it has been anecdotically reported the effectiveness in reversing the effect of diabetes mellitus [40]. However, ozone therapy includes a wide range of administration methods, and more emphasis has been placed on autohemotherapy and its relevant changes in biological targets [20, 33]. We previously demonstrated the statistically significant effect of ozone therapy on periodontal parameters, but no interesting results were obtained regarding glycaemic control [34]. Then, we focused our trial on oxidant/antioxidant balance, by investigating the impact of gaseous ozone therapy add-on periodontal treatment on the oxidative status of diabetic patients suffering periodontitis. In recent years, the ozone therapy has become a very promising therapeutic agent in dentistry [28, 39]. Ozone’s anti-inflammatory, antimicrobial, and antioxidant properties have been largely recognized. Ozone has ability to stimulate 2,3-diphosphoglycerate that in turn induces an elevation in oxygen supply to the tissues, leading to increased production of enzymes, which act as free radical scavengers and cell wall savers, such as superoxide dismutase, glutathione peroxidase, and catalase determining an increase in the output of prostacyclin, which improves vasodilatation and tissue oxygen supply [31]. In our study, diabetic individuals showed low antioxidant defenses. Our results did not confirm protective effects of ozone on endogenous antioxidant defenses. Periodontal parameters improved significantly in Test group, but no statistically significant differences were revealed among oxidative stress markers. To the best of our knowledge, there is no previous evidence to explore the role of gaseous ozone treatment given adjunctive to non-surgical periodontal therapy on oxidative status of diabetic patients with periodontitis prior to our current study. Sagai and Bocci firstly demonstrated ability of ozone to modulate the metabolic pathway of Nrf2. However, this follow-up study not focused on the oxidative stress outcome [49]. The study conducted by Arana et al. on salivary oxidative stress levels in patients with uncontrolled diabetes demonstrated a strong association between oxidative stress and periodontal disease in diabetic conditions [50]. Some studies have demonstrated the antioxidant effect of ozone application in topical form (subgingival ozone irrigation, topical gaseous ozone application) in delaying the progress of periodontitis reporting mixed results, but periodontal use is still poor investigated. Unfortunately, only one study investigated the effect of periodontal treatment and topical application of ozone on plasma levels of reactive oxygen metabolites in diabetic patients affected by periodontitis [13]. Oxidative status of both groups improved significantly after non-surgical periodontal treatment, and at the 3-month evaluation, periodontal parameters decreased significantly for the ozone group. Previous efforts have led to the identification of effector molecules responsible for regulating the biological protective activity of the ozone in the body, as reviewed by Tricarico and Travagli [51]. The mechanism of ozone-tissue interaction is influenced by many factors including the approach used, the conditions of oxidation, and the physical state of the substrate. The gaseous ozone therapy was used to treat periodontal pockets, and local effects were observed. Because of the limited investigational conditions, we do not know what the direct effects of cell homeostasis would be. Our study has some limitations. First, this study was conducted in a single center with a small sample size; second, TAOS, TOS, GSH, and MDA biomarkers were measured at brief-term. Our data did not point out that periodontitis can negatively affect the oxidative status in diabetes, and the effectiveness of periodontal treatment and ozone to our previous works resulted in pointing to metabolic control. The interindividual and intergroup differences may be explainable by different degrees of oxidative stress among the subjects, with distinct capability of activating the Nrf2-dependent oxidative stress metabolic pathway. The higher level of MDA suggest that oxidative stress was not counterbalanced by sufficient antioxidant defences, despite plasma levels of antioxidants were increased.

Although it is demonstrated that periodontitis has a role in development of diabetes, the studies provide an insufficient explanation about the role ROS play in the mechanism at the cellular level. It is required more thorough research.

## Electronic supplementary material

Below is the link to the electronic supplementary material.

Supplementary Material 1

Supplementary Material 2

Supplementary Material 3

## Data Availability

The datasets used and/or analyzed during the current study are available from the corresponding author on reasonable request.
