# Supplementary material for: Research efficacy of gaseous ozone therapy as an adjuvant to periodontal treatment on oxidative stress mediators in patients with type 2 diabetes: a randomized clinical trial
Source: BMC Oral Health. 2023 May 11;23:278. doi: 10.1186/s12903-023-02985-1 (PMC10176779; doi:10.1186/s12903-023-02985-1)
Supplement: Supplementary file 1 — Supplementary Material 1 [file 12903_2023_2985_MOESM1_ESM.docx]

**Appendix 2.**

Homogeneity of Regression Slopes MANCOVA Results:

| Model | Residual df | df | Pillai | F | Numerator df | Denomator df | p |
| --- | --- | --- | --- | --- | --- | --- | --- |
| Interaction Model | 27.000 |  |  |  |  |  |  |
| Original Model | 33.000 | 6.000 | 2.173 | 0.681 | 90.000 | 108.000 | 0.969 |

Covariate-IV Independence

| Covariate | IV | df | Statistic | *p* |
| --- | --- | --- | --- | --- |
| age | severity_of_periodontitis | (1, 36) | 0.180^a^ | 0.674 |
| age | treatment | (1, 36) | 1.612^a^ | 0.212 |
| smoking_status | severity_of_periodontitis | 1 | 1.897^b^ | 0.168 |
| smoking_status | treatment | 1 | 0.245^b^ | 0.621 |
| sex | severity_of_periodontitis | 1 | 1.449^b^ | 0.229 |
| sex | treatment | 1 | 0.622^b^ | 0.430 |
