# Supplementary material for: Research efficacy of gaseous ozone therapy as an adjuvant to periodontal treatment on oxidative stress mediators in patients with type 2 diabetes: a randomized clinical trial
Source: BMC Oral Health. 2023 May 11;23:278. doi: 10.1186/s12903-023-02985-1 (PMC10176779; doi:10.1186/s12903-023-02985-1)
Supplement: Supplementary file 2 — Supplementary Material 2 [file 12903_2023_2985_MOESM2_ESM.pdf]

Appendix 1. Correlations between Dependent Variables

Variable	1	2	3	4	5	6	7	8	9	10	11	12	13	14	15
1. TOS_T0	-														
2. TOS_T1	0.939	-													
3. TOS_T2	0.807	0.905	-												
4. TAS_T1	-0.0707	-0.0658	-0.0736	-											
5. TAS_T2	-0.0951	-0.133	-0.151	0.967	-										
6. TAS_T0	-0.0103	0.0494	0.0542	0.862	0.777	-									
7. BOP_T2	0.114	0.195	0.224	-0.0685	-0.130	0.0275	-								
8. PPD_T2	-0.0650	-0.0191	0.0521	-0.118	-0.151	0.00683	0.223	-							
9. CAL_T2	-0.177	-0.232	-0.277	0.279	0.287	0.242	0.0689	0.394	-						
10. BOP_T0	0.00806	-0.115	-0.188	-0.0235	-0.0117	-0.143	0.297	-0.134	0.0242	-					
11. GSH_T0	-0.00792	-0.0531	0.00920	-0.369	-0.378	-0.356	0.251	0.349	0.0119	0.183	-				
12. CAL_T0	0.288	0.289	0.259	-0.00534	-0.0287	-0.00608	-0.184	-0.150	-0.118	-0.0996	-0.131	-			
13. BOP_T1	0.0214	0.0322	0.0354	-0.104	-0.165	-0.0791	0.576	0.0947	0.0782	0.485	0.0390	-0.0877	-		
14. GSH_T1	0.112	0.0399	-0.0704	-0.0846	-0.0353	-0.0847	0.117	-0.225	0.0431	0.248	-0.227	0.0504	0.00596	-	
15. CAL_T1	-0.345	-0.232	-0.0874	0.302	0.290	0.346	0.177	0.203	0.408	-0.263	-0.283	-0.0919	0.0577	-0.160	-
