# Supplementary material for: Research efficacy of gaseous ozone therapy as an adjuvant to periodontal treatment on oxidative stress mediators in patients with type 2 diabetes: a randomized clinical trial
Source: BMC Oral Health. 2023 May 11;23:278. doi: 10.1186/s12903-023-02985-1 (PMC10176779; doi:10.1186/s12903-023-02985-1)
Supplement: Supplementary file 3 — Supplementary Material 3 [file 12903_2023_2985_MOESM3_ESM.docx]

**Analysis of Covariance. Table for TOS at T0 by severity of periodontitis and treatment while controlling for age, smoking status, and sex.**

Included Variables:
TOS at T0, severity of periodontitis, treatment, age, smoking status, and sex.

**Relationship between TOS at T0 and age:**

| Term | SS | df | F | p |
| --- | --- | --- | --- | --- |
| age | 16.540 | 1 | 0.291 | 0.593 |
| Residuals | 2,158.621 | 38 |  |  |

**Relationship between TOS at T0 and smoking status:**

| Term | SS | df | F | p |
| --- | --- | --- | --- | --- |
| smoking_status | 90.331 | 1 | 1.646 | 0.207 |
| Residuals | 2,084.830 | 38 |  |  |

**Relationship between TOS at T0 and sex:**

| Term | SS | df | F | p |
| --- | --- | --- | --- | --- |
| sex | 0.626 | 1 | 0.0109 | 0.917 |
| Residuals | 2,174.535 | 38 |  |  |

**ANOVA Results:**

| Term | SS | df | F | p | η_p_2 |
| --- | --- | --- | --- | --- | --- |
| severity_of_periodontitis | 35.920 | 1 | 0.625 | 0.435 | 0.0181 |
| treatment | 50.061 | 1 | 0.872 | 0.357 | 0.0250 |
| age | 63.955 | 1 | 1.113 | 0.299 | 0.0317 |
| smoking_status | 86.557 | 1 | 1.507 | 0.228 | 0.0424 |
| sex | 21.400 | 1 | 0.373 | 0.546 | 0.0108 |
| Residuals | 1,952.851 | 34 |  |  |  |

Marginal Means, Standard Error and Sample Size for TOS at T0 by severity of periodontitis and treatment controlling for age, smoking status, and sex:

| Combination | Marginal Means | SE |
| --- | --- | --- |
| M : B | 25.685 | 2.048 |
| S : B | 23.592 | 2.427 |
| M : A | 23.360 | 1.868 |
| S : A | 21.267 | 2.623 |

**Analysis of covariance. Table for TOS at T1 by severity of periodontitis and treatment while controlling for age, smoking status, and sex.**

Included Variables:
TOS at T1, severity of periodontitis, treatment, age, smoking status, and sex.

**Relationship between TOS at T1 and age:**

| Term | SS | df | F | p |
| --- | --- | --- | --- | --- |
| age | 5.719 | 1 | 0.125 | 0.726 |
| Residuals | 1,743.618 | 38 |  |  |

**Relationship between TOS at T1 and smoking status:**

| Term | SS | df | F | p |
| --- | --- | --- | --- | --- |
| smoking_status | 82.680 | 1 | 1.885 | 0.178 |
| Residuals | 1,666.657 | 38 |  |  |

**Relationship between TOS at T1 and sex**:

| Term | SS | df | F | p |
| --- | --- | --- | --- | --- |
| sex | 2.438 | 1 | 0.0530 | 0.819 |
| Residuals | 1,746.899 | 38 |  |  |

**ANOVA Results**:

| Term | SS | df | F | p | η_p_2 |
| --- | --- | --- | --- | --- | --- |
| severity_of_periodontitis | 4.287 | 1 | 0.101 | 0.752 | 0.00296 |
| treatment | 182.883 | 1 | 4.313 | 0.0455 | 0.113 |
| age | 51.724 | 1 | 1.220 | 0.277 | 0.0346 |
| smoking_status | 77.825 | 1 | 1.835 | 0.184 | 0.0512 |
| sex | 13.519 | 1 | 0.319 | 0.576 | 0.00929 |
| Residuals | 1,441.809 | 34 |  |  |  |

Marginal Means, Standard Error and Sample Size for TOS at T1 by severity of periodontitis and treatment controlling for age, smoking status, and sex:

| Combination | Marginal Means | SE |
| --- | --- | --- |
| M : B | 22.742 | 1.760 |
| S : B | 22.019 | 2.085 |
| M : A | 18.298 | 1.605 |
| S : A | 17.575 | 2.254 |

**Analysis of Covariance Table for TOS at T2 by severity of periodontitis and treatment while controlling for age, smoking status, and sex**

Included Variables:
TOS at T2, severity of periodontitis, treatment, age, smoking status, and sex.

**Relationship between TOS at T2 and age:**

| Term | SS | df | F | p |
| --- | --- | --- | --- | --- |
| age | 8.481 | 1 | 0.227 | 0.637 |
| Residuals | 1,422.701 | 38 |  |  |

**Relationship between TOS at T2 and smoking status:**

| Term | SS | df | F | p |
| --- | --- | --- | --- | --- |
| smoking_status | 49.612 | 1 | 1.365 | 0.250 |
| Residuals | 1,381.571 | 38 |  |  |

**Relationship between TOS at T2 and sex:**

| Term | SS | df | F | p |
| --- | --- | --- | --- | --- |
| sex | 5.687 | 1 | 0.152 | 0.699 |
| Residuals | 1,425.495 | 38 |  |  |

ANOVA Results:

| Term | SS | df | F | p | η_p_2 |
| --- | --- | --- | --- | --- | --- |
| severity_of_periodontitis | 0.0118 | 1 | 3.764 × 10^-04^ | 0.985 | 1.107 × 10^-05^ |
| treatment | 262.878 | 1 | 8.370 | 0.00661 | 0.198 |
| age | 59.310 | 1 | 1.888 | 0.178 | 0.0526 |
| smoking_status | 51.576 | 1 | 1.642 | 0.209 | 0.0461 |
| sex | 10.838 | 1 | 0.345 | 0.561 | 0.0100 |
| Residuals | 1,067.861 | 34 |  |  |  |

Marginal Means, Standard Error and Sample Size for TOS at T2 by severity of periodontitis and treatment controlling for age, smoking status, and sex:

| Combination | Marginal Means | SE |
| --- | --- | --- |
| M : B | 19.741 | 1.514 |
| S : B | 19.779 | 1.794 |
| M : A | 14.413 | 1.381 |
| S : A | 14.451 | 1.940 |

**Analysis of Covariance Table for TAS at T1 by severity of periodontitis and treatment while controlling for age, smoking status, and sex**

Included Variables:
TAS at T1, severity of periodontitis, treatment, age, smoking status, and sex.

**Relationship between TAS at T1 and age:**

| Term | SS | df | F | p |
| --- | --- | --- | --- | --- |
| age | 1.745 × 10^-04^ | 1 | 0.00274 | 0.959 |
| Residuals | 2.418 | 38 |  |  |

Relationship between TAS at T1 and smoking status:

| Term | SS | df | F | p |
| --- | --- | --- | --- | --- |
| smoking_status | 0.0661 | 1 | 1.068 | 0.308 |
| Residuals | 2.352 | 38 |  |  |

**Relationship between TAS at T1 and sex:**

| Term | SS | df | F | p |
| --- | --- | --- | --- | --- |
| sex | 0.0418 | 1 | 0.669 | 0.419 |
| Residuals | 2.377 | 38 |  |  |

**ANOVA Results:**

| Term | SS | df | F | p | η_p_2 |
| --- | --- | --- | --- | --- | --- |
| severity_of_periodontitis | 0.0390 | 1 | 0.597 | 0.445 | 0.0172 |
| treatment | 0.0542 | 1 | 0.828 | 0.369 | 0.0238 |
| age | 1.718 × 10^-04^ | 1 | 0.00262 | 0.959 | 7.720 × 10^-05^ |
| smoking_status | 0.0377 | 1 | 0.576 | 0.453 | 0.0167 |
| sex | 0.00477 | 1 | 0.0728 | 0.789 | 0.00214 |
| Residuals | 2.225 | 34 |  |  |  |

Marginal Means, Standard Error and Sample Size for TAS_T1 by severity_of_periodontitis and treatment Controlling for age, smoking_status, and sex:

| Combination | Marginal Means | SE |
| --- | --- | --- |
| M : B | 1.787 | 0.0691 |
| S : B | 1.718 | 0.0819 |
| M : A | 1.864 | 0.0630 |
| S : A | 1.795 | 0.0885 |

**Analysis of Covariance Table for TAS at T2 by severity of periodontitis and treatment while controlling for age, smoking status, and sex**

Included Variables:
TAS at T2, severity of periodontitis, treatment, age, smoking status, and sex.

**Relationship between TAS at T2 and age:**

| Term | SS | df | F | p |
| --- | --- | --- | --- | --- |
| age | 0.00925 | 1 | 0.177 | 0.676 |
| Residuals | 1.981 | 38 |  |  |

**Relationship between TAS at T2 and smoking status:**

| Term | SS | df | F | p |
| --- | --- | --- | --- | --- |
| smoking_status | 0.0279 | 1 | 0.540 | 0.467 |
| Residuals | 1.963 | 38 |  |  |

**Relationship between TAS at T2 and sex:**

| Term | SS | df | F | p |
| --- | --- | --- | --- | --- |
| sex | 0.0715 | 1 | 1.415 | 0.242 |
| Residuals | 1.919 | 38 |  |  |

**ANOVA Results:**

| Term | SS | df | F | p | η_p_2 |
| --- | --- | --- | --- | --- | --- |
| severity_of_periodontitis | 0.0173 | 1 | 0.348 | 0.559 | 0.0101 |
| treatment | 0.172 | 1 | 3.451 | 0.0719 | 0.0921 |
| age | 9.646 × 10^-04^ | 1 | 0.0194 | 0.890 | 5.690 × 10^-04^ |
| smoking_status | 0.0163 | 1 | 0.328 | 0.571 | 0.00955 |
| sex | 0.0197 | 1 | 0.395 | 0.534 | 0.0115 |
| Residuals | 1.694 | 34 |  |  |  |

Marginal Means, Standard Error and Sample Size for TAS at T2 by severity of periodontitis and treatment controlling for age, smoking status, and sex:

| Combination | Marginal Means | SE |
| --- | --- | --- |
| M : B | 1.748 | 0.0603 |
| S : B | 1.702 | 0.0715 |
| M : A | 1.884 | 0.0550 |
| S : A | 1.838 | 0.0773 |

**Analysis of Covariance Table for TAS at T0 by severity of periodontitis and treatment while controlling for age, smoking status, and sex.**

Included Variables:
TAS at T0, severity of periodontitis, treatment, age, smoking status, and sex.

**Relationship between TAS at T0 and age:**

| Term | SS | df | F | p |
| --- | --- | --- | --- | --- |
| age | 0.0150 | 1 | 0.135 | 0.715 |
| Residuals | 4.222 | 38 |  |  |

**Relationship between TAS T0 and smoking status:**

| Term | SS | df | F | p |
| --- | --- | --- | --- | --- |
| smoking_status | 0.0388 | 1 | 0.351 | 0.557 |
| Residuals | 4.198 | 38 |  |  |

**Relationship between TAS T0 and sex:**

| Term | SS | df | F | p |
| --- | --- | --- | --- | --- |
| sex | 0.0583 | 1 | 0.530 | 0.471 |
| Residuals | 4.179 | 38 |  |  |

ANOVA Results:

| Term | SS | df | F | p | η_p_2 |
| --- | --- | --- | --- | --- | --- |
| severity_of_periodontitis | 0.173 | 1 | 1.542 | 0.223 | 0.0434 |
| treatment | 0.192 | 1 | 1.707 | 0.200 | 0.0478 |
| age | 0.0548 | 1 | 0.488 | 0.490 | 0.0141 |
| smoking_status | 0.00154 | 1 | 0.0137 | 0.907 | 4.042 × 10^-04^ |
| sex | 0.0234 | 1 | 0.208 | 0.651 | 0.00608 |
| Residuals | 3.820 | 34 |  |  |  |

Marginal Means, Standard Error, and Sample Size for TAS at T0 by severity of periodontitis and treatment while controlling for age, smoking status, and sex:

| Combination | Marginal Means | SE |
| --- | --- | --- |
| M : B | 1.804 | 0.0906 |
| S : B | 1.659 | 0.107 |
| M : A | 1.660 | 0.0826 |
| S : A | 1.515 | 0.116 |

**Analysis of Covariance Table for GSH at T0 by severity of periodontitis and treatment while controlling for age, smoking status, and sex.**

Included Variables:
GSH at T2, severity of periodontitis, treatment, age, smoking status, and sex.

**Relationship between GSH at T0 and age:**

| Term | SS | df | F | p |
| --- | --- | --- | --- | --- |
| age | 9.121 | 1 | 0.171 | 0.665 |
| Residuals | 1,031.369 | 32 |  |  |

**Relationship between GSH at T0 and sex:**

| Term | SS | df | F | p |
| --- | --- | --- | --- | --- |
| age | 3.519 | 1 | 0.0567 | 0.798 |
| Residuals | 2.023.567 | 36 |  |  |

**Relationship between GSH at T0 and smoking status:**

| Term | SS | df | F | p |
| --- | --- | --- | --- | --- |
| smoking_status | 31.535 | 1 | 0.345 | 0 |
| Residuals | 2.056.345 | 37 |  |  |

**ANOVA Results**

| Term | SS | df | F | p | η_p_2 |
| --- | --- | --- | --- | --- | --- |
| severity_of_periodontitis | 1.162 | 1 | 1.345 | 0.198 | 0.0431 |
| treatment | 0.08896 | 1 | 0.0876 | 0.675 | 0.01418 |
| age | 0.0921 | 1 | 0.0732 | 0.788 | 0.00316 |
| smoking_status | 0.0812 | 1 | 0.103 | 0.750 | 0.02303 |
| sex | 0.0243 | 1 | 0.0256 | 0.876 | 7.079 × 10^-04^ |
| Residuals | 23.203 | 29 |  |  |  |

Marginal Means, Standard Error and Sample Size for GSH at T0 by severity of periodontitis and treatment controlling for age, smoking status, and sex:

| Combination | Marginal Means | SE |
| --- | --- | --- |
| M : B | 1.358 | 0.345 |
| S : B | 1.654 | 0.678 |
| M : A | 1.987 | 0.311 |
| S : A | 1.678 | 0.321 |

**Analysis of Covariance Table for GSH at T1 by severity of periodontitis and treatment while controlling for age, smoking status, and sex**

Included Variables:
GSH at T1, severity of periodontitis, treatment, age, smoking status, and sex.

**Relationship between GSH at T1 and age:**

| Term | SS | df | F | p |
| --- | --- | --- | --- | --- |
| age | 3.453 | 1 | 0.0623 | 0.804 |
| Residuals | 2,050.137 | 37 |  |  |

**Relationship between GSH T1 and smoking status:**

| Term | SS | df | F | p |
| --- | --- | --- | --- | --- |
| smoking_status | 20.669 | 1 | 0.376 | 0.543 |
| Residuals | 2,032.921 | 37 |  |  |

**Relationship between GSH at T1 and sex:**

| Term | SS | df | F | p |
| --- | --- | --- | --- | --- |
| sex | 9.233 | 1 | 0.167 | 0.685 |
| Residuals | 2,044.357 | 37 |  |  |

**ANOVA Results:**

| Term | SS | df | F | p | η_p_2 |
| --- | --- | --- | --- | --- | --- |
| severity_of_periodontitis | 515.345 | 1 | 9.121 | 0.00315 | 0.273 |
| treatment | 158.327 | 1 | 3.231 | 0.0756 | 0.121 |
| age | 14.349 | 1 | 0.342 | 0.478 | 0.00548 |
| smoking_status | 98.509 | 1 | 2.255 | 0.143 | 0.0670 |
| sex | 7.897 | 1 | 0.175 | 0.678 | 0.00784 |
| Residuals | 1,521.317 | 33 |  |  |  |

Marginal Means, Standard Error and Sample Size for GSH at T1 by severity of periodontitis and treatment controlling for age, smoking status, and sex:

| Combination | Marginal Means | SE |
| --- | --- | --- |
| M : B | 19.318 | 1.458 |
| S : B | 8.677 | 2.131 |
| M : A | 14.121 | 1.699 |
| S : A | 5.176 | 1.189 |

**Analysis of Covariance Table for GSH at T2 by severity of periodontitis and treatment while controlling for age, smoking status, and sex**

Included Variables:
GSH at T1, severity of periodontitis, treatment, age, smoking status, and sex.

**Relationship between GSH at T2 and age:**

| Term | SS | df | F | p |
| --- | --- | --- | --- | --- |
| age | 2879 | 1 | 0.0578 | 0.789 |
| Residuals | 1,145.176 | 34 |  |  |

**Relationship between GSH T2 and smoking status:**

| Term | SS | df | F | p |
| --- | --- | --- | --- | --- |
| smoking_status | 19.789 | 1 | 0.675 | 0.487 |
| Residuals | 1,675.934 | 36 |  |  |

**Relationship between GSH at T2 and sex:**

| Term | SS | df | F | p |
| --- | --- | --- | --- | --- |
| sex | 9.233 | 1 | 0.158 | 0.678 |
| Residuals | 2,114.267 | 36 |  |  |

**ANOVA Results:**

| Term | SS | df | F | p | η_p_2 |
| --- | --- | --- | --- | --- | --- |
| severity_of_periodontitis | 512.123 | 1 | 8.678 | 0.00675 | 0.233 |
| treatment | 161.345 | 1 | 3.451 | 0.0856 | 0.231 |
| age | 15.321 | 1 | 0.451 | 0.543 | 0.00518 |
| smoking_status | 97.789 | 1 | 2.345 | 0.151 | 0.0711 |
| sex | 7.678 | 1 | 0.165 | 0.711 | 0.00679 |
| Residuals | 1,611.677 | 36 |  |  |  |

Marginal Means, Standard Error and Sample Size for GSH at T2 by severity of periodontitis and treatment controlling for age, smoking status, and sex:

| Combination | Marginal Means | SE |
| --- | --- | --- |
| M : B | 18.217 | 1.448 |
| S : B | 7.656 | 2.111 |
| M : A | 13.567 | 1.669 |
| S : A | 5.789 | 1.786 |

**Analysis of Covariance Table for MDA at T0 by severity of periodontitis and treatment while controlling for age, smoking status, and sex**

Included Variables:
MDA at T1, severity of periodontitis, treatment, age, smoking status, and sex.

**Relationship between MDA at T0 and age:**

| Term | SS | df | F | p |
| --- | --- | --- | --- | --- |
| age | 3.453 | 1 | 0.0623 | 0.804 |
| Residuals | 2,050.137 | 37 |  |  |

**Relationship between MDA T0 and smoking status:**

| Term | SS | df | F | p |
| --- | --- | --- | --- | --- |
| smoking_status | 20.669 | 1 | 0.376 | 0.543 |
| Residuals | 2,032.921 | 37 |  |  |

**Relationship between MDA at T0 and sex:**

| Term | SS | df | F | p |
| --- | --- | --- | --- | --- |
| sex | 9.233 | 1 | 0.167 | 0.685 |
| Residuals | 2,044.357 | 37 |  |  |

**ANOVA Results:**

| Term | SS | df | F | p | η_p_2 |
| --- | --- | --- | --- | --- | --- |
| severity_of_periodontitis | 456.345 | 1 | 9.121 | 0.00315 | 0.273 |
| treatment | 161.317 | 1 | 2.7896 | 0.0578 | 0.132 |
| age | 15.123 | 1 | 0.547 | 0.765 | 0.00748 |
| smoking_status | 95.501 | 1 | 2.255 | 0.129 | 0.0876 |
| sex | 7.897 | 1 | 0.156 | 0.698 | 0.00794 |
| Residuals | 1,531.327 | 33 |  |  |  |

Marginal Means, Standard Error and Sample Size for MDA at T0 by severity of periodontitis and treatment controlling for age, smoking status, and sex:

| Combination | Marginal Means | SE |
| --- | --- | --- |
| M : B | 20.132 | 1.768 |
| S : B | 7.897 | 1.988 |
| M : A | 14.121 | 1.675 |
| S : A | 4.799 | 1.454 |

**Analysis of Covariance Table for MDA at T1 by severity of periodontitis and treatment while controlling for age, smoking status, and sex**

Included Variables:
GSH at T1, severity of periodontitis, treatment, age, smoking status, and sex.

**Relationship between MDA at T1 and age:**

| Term | SS | df | F | p |
| --- | --- | --- | --- | --- |
| age | 2.893 | 1 | 0.0613 | 0.998 |
| Residuals | 2,089.131 | 36 |  |  |

**Relationship between MDA T1 and smoking status:**

| Term | SS | df | F | p |
| --- | --- | --- | --- | --- |
| smoking_status | 12.564 | 1 | 0.447 | 0.519 |
| Residuals | 2,045.787 | 37 |  |  |

**Relationship between MDA at T1 and sex:**

| Term | SS | df | F | p |
| --- | --- | --- | --- | --- |
| sex | 9.233 | 1 | 0.167 | 0.685 |
| Residuals | 1,949.787 | 39 |  |  |

**ANOVA Results:**

| Term | SS | df | F | p | η_p_2 |
| --- | --- | --- | --- | --- | --- |
| severity_of_periodontitis | 213.366 | 1 | 8.577 | 0.00389 | 0.365 |
| treatment | 178.577 | 1 | 3.231 | 0.0756 | 0.117 |
| age | 17.189 | 1 | 0.342 | 0.478 | 0.00456 |
| smoking_status | 88.657 | 1 | 2.576 | 0.435 | 0.0455 |
| sex | 6.889 | 1 | 0.469 | 0.788 | 0.00714 |
| Residuals | 1,345.654 | 31 |  |  |  |

Marginal Means, Standard Error and Sample Size for MDA at T1 by severity of periodontitis and treatment controlling for age, smoking status, and sex:

| Combination | Marginal Means | SE |
| --- | --- | --- |
| M : B | 21.091 | 1.376 |
| S : B | 8.986 | 2.112 |
| M : A | 15.766 | 1.879 |
| S : A | 5.176 | 1.189 |

**Analysis of Covariance Table for MDA at T2 by severity of periodontitis and treatment while controlling for age, smoking status, and sex**

Included Variables:
MDA at T2, severity of periodontitis, treatment, age, smoking status, and sex.

**Relationship between MDA at T2 and age:**

| Term | SS | df | F | p |
| --- | --- | --- | --- | --- |
| age | 4.123 | 1 | 0.5678 | 0.780 |
| Residuals | 2,111.156 | 32 |  |  |

**Relationship between MDA T2 and smoking status:**

| Term | SS | df | F | p |
| --- | --- | --- | --- | --- |
| smoking_status | 20.669 | 1 | 0.376 | 0.543 |
| Residuals | 2,032.921 | 37 |  |  |

**Relationship between MDA at T2 and sex:**

| Term | SS | df | F | p |
| --- | --- | --- | --- | --- |
| sex | 10.123 | 1 | 0.199 | 0.711 |
| Residuals | 1.056.367 | 35 |  |  |

**ANOVA Results:**

| Term | SS | df | F | p | η_p_2 |
| --- | --- | --- | --- | --- | --- |
| severity_of_periodontitis | 610.312 | 1 | 8.6779 | 0.00031 | 0.311 |
| treatment | 161.676 | 1 | 2.897 | 0.0567 | 0.132 |
| age | 15.789 | 1 | 0.312 | 0.456 | 0.00117 |
| smoking_status | 94.686 | 1 | 2.255 | 0.143 | 0.0593 |
| sex | 8.212 | 1 | 0.569 | 0.883 | 0.00761 |
| Residuals | 1,555.311 | 33 |  |  |  |

Marginal Means, Standard Error and Sample Size for MDA at T2 by severity of periodontitis and treatment controlling for age, smoking status, and sex:

| Combination | Marginal Means | SE |
| --- | --- | --- |
| M : B | 21.311 | 1.438 |
| S : B | 8.677 | 2.123 |
| M : A | 13.984 | 1.664 |
| S : A | 5.236 | 1.564 |

**Analysis of Covariance Table for BOP at T2 by severity of periodontitis and treatment while controlling for age, smoking status, and sex**

Included Variables:
BOP T2, severity of periodontitis, treatment, age, smoking status, and sex.

**Relationship between BOP at T2 and age:**

| Term | SS | df | F | p |
| --- | --- | --- | --- | --- |
| age | 3.453 | 1 | 0.0623 | 0.804 |
| Residuals | 2,050.137 | 37 |  |  |

**Relationship between BOP T2 and smoking status:**

| Term | SS | df | F | p |
| --- | --- | --- | --- | --- |
| smoking_status | 20.669 | 1 | 0.376 | 0.543 |
| Residuals | 2,032.921 | 37 |  |  |

**Relationship between BOP T2 and sex:**

| Term | SS | df | F | p |
| --- | --- | --- | --- | --- |
| sex | 9.233 | 1 | 0.167 | 0.685 |
| Residuals | 2,044.357 | 37 |  |  |

**ANOVA Results:**

| Term | SS | df | F | p | η_p_2 |
| --- | --- | --- | --- | --- | --- |
| severity_of_periodontitis | 485.355 | 1 | 11.113 | 0.00213 | 0.252 |
| treatment | 161.319 | 1 | 3.694 | 0.0633 | 0.101 |
| age | 13.789 | 1 | 0.316 | 0.578 | 0.00948 |
| smoking_status | 98.509 | 1 | 2.255 | 0.143 | 0.0640 |
| sex | 7.307 | 1 | 0.167 | 0.685 | 0.00504 |
| Residuals | 1,441.319 | 33 |  |  |  |

Marginal Means, Standard Error and Sample Size for BOP at T2 by severity of periodontitis and treatment controlling for age, smoking status, and sex:

| Combination | Marginal Means | SE |
| --- | --- | --- |
| M : B | 17.253 | 1.798 |
| S : B | 9.397 | 2.131 |
| M : A | 13.010 | 1.639 |
| S : A | 5.155 | 2.380 |

**Analysis of Covariance Table for PPD at T2 by severity of periodontitis and treatment while controlling for age, smoking status, and sex.**

Included Variables:
PPD at T2, severity of periodontitis, treatment, age, smoking status, and sex.

**Relationship between PPD at T2 and age:**

| Term | SS | df | F | p |
| --- | --- | --- | --- | --- |
| age | 0.127 | 1 | 0.174 | 0.679 |
| Residuals | 27.901 | 38 |  |  |

**Relationship between PPD at T2 and smoking status:**

| Term | SS | df | F | p |
| --- | --- | --- | --- | --- |
| smoking_status | 1.100 × 10^-04^ | 1 | 1.491 × 10^-04^ | 0.990 |
| Residuals | 28.028 | 38 |  |  |

**Relationship between PPD at T2 and sex:**

| Term | SS | df | F | p |
| --- | --- | --- | --- | --- |
| sex | 0.00435 | 1 | 0.00590 | 0.939 |
| Residuals | 28.024 | 38 |  |  |

**ANOVA Results:**

| Term | SS | df | F | p | η_p_2 |
| --- | --- | --- | --- | --- | --- |
| severity_of_periodontitis | 1.178 | 1 | 1.500 | 0.229 | 0.0423 |
| treatment | 0.0609 | 1 | 0.0776 | 0.782 | 0.00228 |
| age | 0.0575 | 1 | 0.0732 | 0.788 | 0.00215 |
| smoking_status | 0.0812 | 1 | 0.103 | 0.750 | 0.00303 |
| sex | 0.0243 | 1 | 0.0310 | 0.861 | 9.099 × 10^-04^ |
| Residuals | 26.705 | 34 |  |  |  |

Marginal Means, Standard Error and Sample Size for PPD at T2 by severity of periodontitis and treatment controlling for age, smoking status, and sex:

| Combination | Marginal Means | SE |
| --- | --- | --- |
| M : B | 2.158 | 0.239 |
| S : B | 1.779 | 0.284 |
| M : A | 2.077 | 0.218 |
| S : A | 1.698 | 0.307 |

**Analysis of Covariance Table for CAL at T2 by severity of periodontitis and treatment while controlling for age, smoking status, and sex.**

Included Variables:
CAL at T2, severity of periodontitis, smoking status, age, and sex.

**Relationship between CAL at T2 and age:**

| Term | SS | df | F | p |
| --- | --- | --- | --- | --- |
| age | 0.144 | 1 | 0.130 | 0.721 |
| Residuals | 42.225 | 38 |  |  |

Relationship between CAL at T2 and smoking status:

| Term | SS | df | F | p |
| --- | --- | --- | --- | --- |
| smoking_status | 0.760 | 1 | 0.694 | 0.410 |
| Residuals | 41.610 | 38 |  |  |

**Relationship between CAL at T2 and sex:**

| Term | SS | df | F | p |
| --- | --- | --- | --- | --- |
| sex | 3.445 | 1 | 3.363 | 0.0745 |
| Residuals | 38.925 | 38 |  |  |

**ANOVA Results:**

| Term | SS | df | F | p | η_p_2 |
| --- | --- | --- | --- | --- | --- |
| severity_of_periodontitis | 3.927 | 1 | 4.878 | 0.0340 | 0.125 |
| treatment | 5.852 | 1 | 7.269 | 0.0108 | 0.176 |
| age | 0.575 | 1 | 0.715 | 0.404 | 0.0206 |
| smoking_status | 0.0154 | 1 | 0.0191 | 0.891 | 5.629 × 10^-04^ |
| sex | 1.224 | 1 | 1.521 | 0.226 | 0.0428 |
| Residuals | 27.371 | 34 |  |  |  |

Marginal Means, Standard Error and Sample Size for CAL at T2 by severity of periodontitis, smoking status, age, and sex:

| Combination | Marginal Means | SE |
| --- | --- | --- |
| M : B | 2.497 | 0.242 |
| S : B | 1.805 | 0.287 |
| M : A | 3.292 | 0.221 |
| S : A | 2.600 | 0.311 |

**Analysis of Covariance Table for BOP at T0 by severity of periodontitis and treatment while controlling for age, smoking status, and sex.**

Included Variables:
BOP at T0, severity of periodontitis, treatment, age, smoking status, and sex.

**Relationship between BOP at T0 and age:**

| Term | SS | df | F | p |
| --- | --- | --- | --- | --- |
| age | 174.934 | 1 | 0.291 | 0.593 |
| Residuals | 22,248.502 | 37 |  |  |

**Relationship between BOP at T0 and smoking status:**

| Term | SS | df | F | p |
| --- | --- | --- | --- | --- |
| smoking_status | 1.984 | 1 | 0.00327 | 0.955 |
| Residuals | 22,421.452 | 37 |  |  |

**Relationship between BOP at T0 and sex:**

| Term | SS | df | F | p |
| --- | --- | --- | --- | --- |
| sex | 0.339 | 1 | 5.590 × 10^-04^ | 0.981 |
| Residuals | 22,423.097 | 37 |  |  |

**ANOVA Results:**

| Term | SS | df | F | p | η_p_2 |
| --- | --- | --- | --- | --- | --- |
| severity_of_periodontitis | 2,307.453 | 1 | 4.321 | 0.0455 | 0.116 |
| treatment | 1,580.580 | 1 | 2.960 | 0.0947 | 0.0823 |
| age | 293.257 | 1 | 0.549 | 0.464 | 0.0164 |
| smoking_status | 36.206 | 1 | 0.0678 | 0.796 | 0.00205 |
| sex | 99.585 | 1 | 0.186 | 0.669 | 0.00562 |
| Residuals | 17,624.111 | 33 |  |  |  |

Marginal Means, Standard Error and Sample Size for BOP at T0 by severity of periodontitis, smoking status, age, and sex:

| Combination | Marginal Means | SE |
| --- | --- | --- |
| M : B | 63.246 | 6.287 |
| S : B | 46.118 | 7.451 |
| M : A | 76.527 | 5.733 |
| S : A | 59.399 | 8.321 |

**Analysis of Covariance Table for PPD at T0 by severity of periodontitis and treatment while controlling for age, smoking status, and sex**

Included Variables:
PPD at T0, severity of periodontitis, smoking status, age, and sex.

**Relationship between PPD at T0 and age:**

| Term | SS | df | F | p |
| --- | --- | --- | --- | --- |
| age | 4.258 | 1 | 3.216 | 0.0809 |
| Residuals | 50.320 | 38 |  |  |

**Relationship between PPD at T0 and smoking status:**

| Term | SS | df | F | p |
| --- | --- | --- | --- | --- |
| smoking_status | 0.00505 | 1 | 0.00351 | 0.953 |
| Residuals | 54.573 | 38 |  |  |

Relationship between PPD_T0 and sex:

| Term | SS | df | F | p |
| --- | --- | --- | --- | --- |
| sex | 0.140 | 1 | 0.0980 | 0.756 |
| Residuals | 54.438 | 38 |  |  |

**ANOVA Results:**

| Term | SS | df | F | p | η_p_2 |
| --- | --- | --- | --- | --- | --- |
| severity_of_periodontitis | 0.775 | 1 | 0.534 | 0.470 | 0.0155 |
| treatment | 1.609 × 10^-09^ | 1 | 1.109 × 10^-09^ | 1.000 | 3.262 × 10^-11^ |
| age | 3.938 | 1 | 2.715 | 0.109 | 0.0739 |
| smoking_status | 0.316 | 1 | 0.218 | 0.644 | 0.00636 |
| sex | 0.0392 | 1 | 0.0270 | 0.870 | 7.943 × 10^-04^ |
| Residuals | 49.321 | 34 |  |  |  |

Marginal Means, Standard Error and Sample Size for PPD at T0 by severity of periodontitis, smoking status, age, and sex:

| Combination | Marginal Means | SE |
| --- | --- | --- |
| M : B | 4.578 | 0.325 |
| S : B | 4.271 | 0.386 |
| M : A | 4.578 | 0.297 |
| S : A | 4.271 | 0.417 |

**Analysis of Covariance Table for CAL at T0 by severity of periodontitis and treatment while controlling for age, smoking status, and sex.**

Included Variables:
CAL at T0, severity of periodontitis, smoking status, age, and sex:

**Relationship between CAL at T0 and age:**

| Term | SS | df | F | p |
| --- | --- | --- | --- | --- |
| age | 0.228 | 1 | 0.435 | 0.514 |
| Residuals | 19.926 | 38 |  |  |

**Relationship between CAL at T0 and smoking status:**

| Term | SS | df | F | p |
| --- | --- | --- | --- | --- |
| smoking_status | 0.0121 | 1 | 0.0228 | 0.881 |
| Residuals | 20.142 | 38 |  |  |

**Relationship between CAL at T0 and sex:**

| Term | SS | df | F | p |
| --- | --- | --- | --- | --- |
| sex | 0.0582 | 1 | 0.110 | 0.742 |
| Residuals | 20.096 | 38 |  |  |

**ANOVA Results:**

| Term | SS | df | F | p | η_p_2 |
| --- | --- | --- | --- | --- | --- |
| severity_of_periodontitis | 1.861 | 1 | 3.518 | 0.0693 | 0.0938 |
| treatment | 0.00512 | 1 | 0.00967 | 0.922 | 2.843 × 10^-04^ |
| age | 0.326 | 1 | 0.615 | 0.438 | 0.0178 |
| smoking_status | 0.0114 | 1 | 0.0216 | 0.884 | 6.346 × 10^-04^ |
| sex | 0.00423 | 1 | 0.00800 | 0.929 | 2.353 × 10^-04^ |
| Residuals | 17.992 | 34 |  |  |  |

Marginal Means, Standard Error and Sample Size for CAL at T0 by severity of periodontitis, smoking status, age, and sex:

| Combination | Marginal Means | SE |
| --- | --- | --- |
| M : B | 5.259 | 0.197 |
| S : B | 5.735 | 0.233 |
| M : A | 5.282 | 0.179 |
| S : A | 5.759 | 0.252 |

**Analysis of Covariance Table for BOP at T1 by severity of periodontitis and treatment while controlling for age, smoking status, and sex.**

Included Variables:
BOP at T1, severity of periodontitis, smoking status, age, and sex.

**Relationship between BOP at T1 and age:**

| Term | SS | df | F | p |
| --- | --- | --- | --- | --- |
| age | 190.367 | 1 | 2.558 | 0.118 |
| Residuals | 2,753.992 | 37 |  |  |

**Relationship between BOP at T1 and smoking status:**

| Term | SS | df | F | p |
| --- | --- | --- | --- | --- |
| smoking_status | 27.438 | 1 | 0.348 | 0.559 |
| Residuals | 2,916.921 | 37 |  |  |

**Relationship between BOP at T1 and sex:**

| Term | SS | df | F | p |
| --- | --- | --- | --- | --- |
| sex | 50.105 | 1 | 0.641 | 0.429 |
| Residuals | 2,894.254 | 37 |  |  |

ANOVA Results:

| Term | SS | df | F | p | η_p_2 |
| --- | --- | --- | --- | --- | --- |
| severity_of_periodontitis | 182.445 | 1 | 2.396 | 0.131 | 0.0677 |
| treatment | 0.300 | 1 | 0.00394 | 0.950 | 1.195 × 10^-04^ |
| age | 117.784 | 1 | 1.547 | 0.222 | 0.0448 |
| smoking_status | 6.041 | 1 | 0.0793 | 0.780 | 0.00240 |
| sex | 75.887 | 1 | 0.997 | 0.325 | 0.0293 |
| Residuals | 2,512.569 | 33 |  |  |  |

Marginal Means, Standard Error and Sample Size for BOP at T1 by severity of periodontitis, smoking status, age, and sex:

| Combination | Marginal Means | SE |
| --- | --- | --- |
| M : B | 22.161 | 2.374 |
| S : B | 17.345 | 2.813 |
| M : A | 21.978 | 2.165 |
| S : A | 17.162 | 3.142 |

**Analysis of Covariance Table for PPD at T1 by severity of periodontitis and treatment while controlling for age, smoking status, and sex**

Included Variables:
PPD at T1, severity of periodontitis, smoking status, age, and sex.

**Relationship between PPD at T1 and age:**

| Term | SS | df | F | p |
| --- | --- | --- | --- | --- |
| age | 20.877 | 1 | 2.079 | 0.158 |
| Residuals | 381.676 | 38 |  |  |

Relationship between PPD at T1 and smoking status:

| Term | SS | df | F | p |
| --- | --- | --- | --- | --- |
| smoking_status | 15.004 | 1 | 1.471 | 0.233 |
| Residuals | 387.550 | 38 |  |  |

**Relationship between PPD at T1 and sex:**

| Term | SS | df | F | p |
| --- | --- | --- | --- | --- |
| sex | 11.416 | 1 | 1.109 | 0.299 |
| Residuals | 391.137 | 38 |  |  |

**ANOVA Results:**

| Term | SS | df | F | p | η_p_2 |
| --- | --- | --- | --- | --- | --- |
| severity_of_periodontitis | 22.326 | 1 | 2.395 | 0.131 | 0.0658 |
| treatment | 17.972 | 1 | 1.928 | 0.174 | 0.0537 |
| age | 14.574 | 1 | 1.563 | 0.220 | 0.0440 |
| smoking_status | 5.796 | 1 | 0.622 | 0.436 | 0.0180 |
| sex | 17.430 | 1 | 1.870 | 0.180 | 0.0521 |
| Residuals | 316.979 | 34 |  |  |  |

Marginal Means, Standard Error and Sample Size for PPD at T1 by severity of periodontitis, smoking status, age, and sex:

| Combination | Marginal Means | SE |
| --- | --- | --- |
| M : B | 2.625 | 0.825 |
| S : B | 0.975 | 0.978 |
| M : A | 4.018 | 0.753 |
| S : A | 2.368 | 1.057 |

**Analysis of Covariance Table for CAL at T1 by severity of periodontitis and treatment while controlling for age, smoking status, and sex**

Included Variables:
CAL at T1, severity of periodontitis, smoking status, age, and sex.

**Relationship between CAL at T1 and age:**

| Term | SS | df | F | p |
| --- | --- | --- | --- | --- |
| age | 0.947 | 1 | 1.006 | 0.322 |
| Residuals | 35.765 | 38 |  |  |

**Relationship between CAL at T1 and smoking status:**

| Term | SS | df | F | p |
| --- | --- | --- | --- | --- |
| smoking_status | 0.0126 | 1 | 0.0130 | 0.910 |
| Residuals | 36.700 | 38 |  |  |

**Relationship between CAL at T1 and sex:**

| Term | SS | df | F | p |
| --- | --- | --- | --- | --- |
| sex | 1.104 | 1 | 1.178 | 0.285 |
| Residuals | 35.609 | 38 |  |  |

**ANOVA Results:**

| Term | SS | df | F | p | η_p_2 |
| --- | --- | --- | --- | --- | --- |
| severity_of_periodontitis | 3.410 | 1 | 3.881 | 0.0570 | 0.102 |
| treatment | 1.505 | 1 | 1.713 | 0.199 | 0.0480 |
| age | 0.617 | 1 | 0.702 | 0.408 | 0.0202 |
| smoking_status | 0.765 | 1 | 0.871 | 0.357 | 0.0250 |
| sex | 1.093 | 1 | 1.244 | 0.272 | 0.0353 |
| Residuals | 29.870 | 34 |  |  |  |

Marginal Means, Standard Error and Sample Size for CAL at T1 by severity of periodontitis, smoking status, age, and sex:

| Combination | Marginal Means | SE |
| --- | --- | --- |
| M : B | 3.474 | 0.253 |
| S : B | 2.829 | 0.300 |
| M : A | 3.071 | 0.231 |
| S : A | 2.426 | 0.324 |
